# Characterization of the complete plastome of *Delphinium montanum*, a polyploid, endemic and endangered Pyrenean Larkspur

**DOI:** 10.1080/23802359.2022.2057248

**Published:** 2022-04-03

**Authors:** Pascaline Salvado, Christel Llauro, Marie-Christine Carpentier, Valérie Delorme-Hinoux, Joris A. M. Bertrand

**Affiliations:** Laboratoire Génome & Développement des Plantes (UMR 5096 UPVD/CNRS), University of Perpignan Via Domitia, Perpignan, France

**Keywords:** *Delphinium montanum*, Pyrenean Larkspur, Ranunculaceae, chloroplast (cp) genome, Illumina sequencing, *Nanopore sequencing*

## Abstract

*Delphinium montanum* DC. 1815, is an endangered larkspur endemic to the Eastern Pyrenees. For biogeographic and conservation purpose, a hybrid assembly approach based on long- and short-read genomic data allowed us to successfully assemble whole plastid genome of *Delphinium montanum*. The complete plastome is 154,185 bp in length, consisting of a pair of inverted repeats (IRs) of 26,559 bp, a large single-copy (LSC) region and a small single-copy region (SSC) of 84,746 and 16,320 bp, respectively. It was found to contain 136 genes, including 84 protein-coding genes, 44 trRNA genes and 8 rRNA genes. The overall GC content of the plastid genome is 38.3%. Phylogenetic inference supports the polyphyly of the *Delphinium* genus.

*Delphinium montanum* DC. 1815 (Ranunculaceae), is a perennial larkspur endemic to the Eastern part of the Pyrenees on both the Spanish and French sides. Its range is restricted to high mountain ecosystems (1600–2400 m), and it is now only observable at a dozen of localities. These populations display deficit in heterozygotes and a high degree of genetic structure (Simon et al. 2001; López-Pujol et al. 2007; Salvado et al. 2021). This species represents a particular conservation issue in a context of global change as its populations are doomed to adapt or die on their ‘sky islands’.

We used an adapted CTAB method (see Supplementary online materials) to extract genomic DNA from an individual of *Delphinium montanum* collected near Orri de Baix, France (N 42.445697° E 2.12259°) with appropriate permit (‘Arrêté préfectoral n°13616*01’ issued by the ‘Direction Départementale des Territoires et de la Mer 66' (DDTM 66), on 26-May-2020) and deposited in the collection of the University of Perpignan Via Domitia (www.univ-perp.fr, J. Bertrand, joris.bertrand@univ-perp.fr) under accession 21-Dmo-006. We used a hybrid assembly approach based on short-read (Illumina) and long-read (Oxford Nanopore Technologies) sequencing (see Supplementary online materials and Bertrand et al. 2021 for details). In brief, we used Unicycler v0.4.9b (Wick et al. 2017) to reconstruct plastid genome, the web-based interface of GeSeq (Tillich et al. 2017) to carry on gene annotation, as well as the viewing and editing features of Geneious v.11.0.5 (www.geneious.com). The sequence is available from GenBank (Accession no.: OK148444).

The plastid genome of *D. montanum* is a circular molecule of 154,185 bp in length, comprising a large single-copy (LSC) region and a small single-copy region (SSC) of 84,746 and 16,320 bp, respectively, separated by two inverted repeat regions (IR) of 26,559 bp. We annotated 113 distinct genes, including 77 protein-coding genes, 4 ribosomal RNA genes (all located in the IRs) and 32 distinct tRNA genes. The genome contained 92 unique genes, 18 genes duplicated in the IRs, two (*trn*E-UUC, *trn*M-CAU) triplicated genes in the LSC and one partially duplicated gene split between LSC and IRs (*rps*12). Among annotated genes, 8 contained one intron (*atp*F, *ndh*A, *ndh*B, *pet*B, *pet*D, *rpl*2, *rpl*16, *rpo*C1) and 3 contained two introns (*clp*P1, *paf*I, *rps*12). The overall GC content of 38.3% was of 36.4, 32.8 and 43.0% in the LSC, SSC and IR regions respectively (see Supplementary online materials for more details).

The *Delphinium montanum* plastid genome was comparable in size and structure to 8 other published plastomes of *Delphinium* species. We used MAFFT v7.3.88 (Katoh et al. 2002; Katoh and Standley 2013) to align the plastome of *D. montanum* with a set of other previously published plastid genomes of the Delphinieae tribe. We then reconstructed a phylogenetic tree to verify its systematics placement with IQ-TREE v2.0.6 (Minh et al. 2020) with 1000 replicates (-B 1000) of Ultrafast Bootstrap Approximation (UFBoot) to assess nodes support. As previously shown, *D. montanum* is part of the *Delphinium* subgenus *Delphinastrum* (Jabbour and Renner 2012). Our data also supports that *Delphinium* is a non-monophyletic genus (Figure 1).

**Figure 1.**
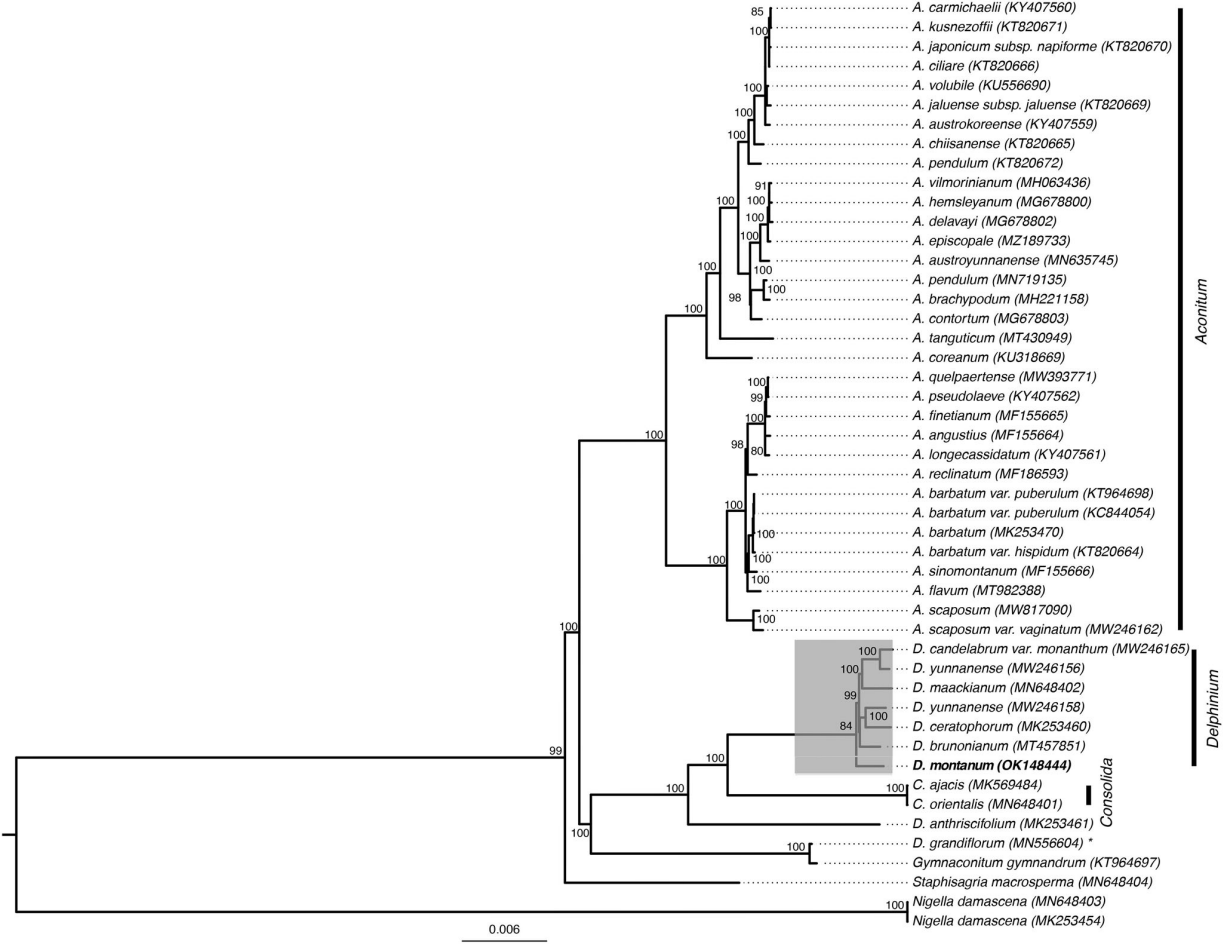
Phylogenetic position of *D. montanum* inferred by Maximum Likelihood method based on 8 whole-plastome *Delphinium* sequences. Node supports correspond to bootstrap values.

## Data Availability

The consensus genome sequence is openly available in GenBank of NCBI at [https://www.ncbi.nlm.nih.gov] (https://www.ncbi.nlm.nih.gov/) under the accession no. OK148444. The genomic data that support the findings of this study are available from the European Nucleotide Archive at [https://www.ebi.ac.uk/ena/] (https://www.ebi.ac.uk/ena/) The associated numbers are Study: PRJEB46773, Sample: ERS7180281, and Runs: ERR6447361 (Illumina) and ERR6447534/ERR6447760 (ONT), respectively.
